# Myeloid cells as potential targets for immunotherapy in pediatric gliomas

**DOI:** 10.3389/fped.2024.1346493

**Published:** 2024-03-08

**Authors:** Stephen C. Frederico, Nikhil Sharma, Corbin Darling, Suchet Taori, Alexandra C. Dubinsky, Xiaoran Zhang, Itay Raphael, Gary Kohanbash

**Affiliations:** ^1^ University of Pittsburgh School of Medicine, Pittsburgh, PA, United States; ^2^ Harvard Medical School, Boston, MA, United States; ^3^ Dana-Farber Cancer Institute, Boston, MA, United States; ^4^ Department of Neurological Surgery, University of Pittsburgh, Pittsburgh, PA, United States; ^5^ Sloan Kettering Memorial Cancer Center, New York, NY, United States; ^6^ Department of Immunology, University of Pittsburgh, Pittsburgh, PA, United States

**Keywords:** glioblastoma, HGG, immunotherapy, myeloid, brain, pediatric, cancer, glioma

## Abstract

Pediatric high-grade glioma (pHGG) including pediatric glioblastoma (pGBM) are highly aggressive pediatric central nervous system (CNS) malignancies. pGBM comprises approximately 3% of all pediatric CNS malignancies and has a 5-year survival rate of approximately 20%. Surgical resection and chemoradiation are often the standard of care for pGBM and pHGG, however, even with these interventions, survival for children diagnosed with pGBM and pHGG remains poor. Due to shortcomings associated with the standard of care, many efforts have been made to create novel immunotherapeutic approaches targeted to these malignancies. These efforts include the use of vaccines, cell-based therapies, and immune-checkpoint inhibitors. However, it is believed that in many pediatric glioma patients an immunosuppressive tumor microenvironment (TME) possess barriers that limit the efficacy of immune-based therapies. One of these barriers includes the presence of immunosuppressive myeloid cells. In this review we will discuss the various types of myeloid cells present in the glioma TME, including macrophages and microglia, myeloid-derived suppressor cells, and dendritic cells, as well as the specific mechanisms these cells can employ to enable immunosuppression. Finally, we will highlight therapeutic strategies targeted to these cells that are aimed at impeding myeloid-cell derived immunosuppression.

## Introduction

Brain and spinal cord tumors are the most common solid tumor and a leading cause of cancer-related death in children. Currently, brain and spinal cord tumors account for approximately 25% of childhood cancers (1). Of brain tumors that arise in children, 8%–10% of these tumors are high-grade gliomas (2). Pediatric high-grade gliomas (pHGGs) have a poor prognosis with a median overall survival of less than 2 years from initial diagnosis (2, 3). In adults, a majority of high-grade gliomas occur in supratentorial regions, while in children they occur at a higher frequency in infratentorial regions such as the cerebellum, brainstem, and thalamus (4). pHGGs can be divided into three main molecular subtypes those being histone H3 mutant, isocitrate dehydrogenase gene (IDH) mutant, and H3/IDH wildtype (5). Other types include infant-type hemispheric glioma, and epidermal growth factor receptor (EGFR) mutant or ACVR mutant—diffuse midline glioma (DMG) (5, 6).

The discovery of histone H3 somatic mutations was pivotal for the field of pediatric neuro-oncology as this finding provided insights into the unique biology and underpinning of several pHGGs (7–11). H3 somatic mutations result in amino acid substitutions (12). While H3.3K27-mutant pHGG is a more common H3 mutant pHGG, other variants include, H3.1K27-mutant and H3.2K27-mutant pHGG, H3-wildtype with EZH Inhibitory Protein (EZHIP) overexpression pHGG, EGFR-altered pHGG, and H3 G34-mutant pHGG (13, 14). One pHGG that resides outside the H3 mutant molecular subtype includes pediatric glioblastoma (pGBM) (15). Glioblastoma is the most common and lethal of all primary brain tumors, however, this is predominately a disease that affects older adults (16–21). pGBM is considered a rare tumor in children as this tumor accounts for approximately 3% of all pediatric central nervous system (CNS) malignancies (16). pGBM is a highly aggressive malignancy as the median survival for children diagnosed with pGBM is 13–73 months (22). The 5-year survival rate for children diagnosed with pGBM is approximately 20% (22).

Surgery and chemoradiation, when safe, are considered the standard of care for pediatric gliomas, including pGBM (23). However, despite these interventions, outcomes for children diagnosed with pGBM remains poor (23). There have been numerous clinical trials aimed at using immunotherapy to combat pediatric gliomas (24, 25). However, immunotherapy based clinical trials for the treatment of pediatric gliomas have yet to demonstrate robust therapeutic efficacy (26). There are several barriers that are believed to impede immunotherapy efficacy in patients with pediatric gliomas. A major barrier of successful immunotherapy is the increased presence of infiltrating myeloid cells in the glioma TME (27); Myeloid cells can be abundant in the TME of adult and pediatric gliomas, are largely considered immunosuppressive, and exhibit a wide spectrum of phenotypes (26, 28–30). Myeloid cells originate from a common progenitor cell developed from hematopoietic stem cells in the bone marrow (31). Additionally, microglia, which are brain-resident macrophages, are derived from yolk sac erythro-myeloid progenitors during development (31, 32). Common myeloid progenitor cells can give rise to megakaryocytes, erythrocytes, mast cells, or myeloblasts (33, 34). Myeloblasts further differentiate into basophils, eosinophils, neutrophils, and monocytes further can mature into subsets of macrophages or dendritic cells (33, 34).

Although little is known about the function of myeloid cells in pediatric glioma, emerging evidence suggests that these cells are abundant in the TME of pediatric gliomas, including pGBM, and may be associated with patient outcomes and response to immunotherapy (27, 35, 36). In this review we will highlight the different myeloid cell populations that are known to be present in pediatric gliomas, as well as other myeloid cell types that we hypothesize are in the pediatric glioma TME, due to their established presence in adult gliomas. We will discuss the specific mechanisms these cells employ to enable immune suppression (Table 1). Due to the paucity of studies discussing the mechanisms myeloid cells use to induce immune suppression specifically in pGBM, many of the myeloid-related immunosuppressive mechanisms that we will highlight in this manuscript have been observed in adult gliomas as well as non-CNS tumors. We will also discuss potential therapies that are being targeted to these cell populations as this may help in reducing the immunosuppressive nature of pGBM and improve immunotherapy efficacy.

**Table 1:** Myeloid cells present in the glioma microenvironment.

Cell type	Major surface markers	Primary cytokines released	Effects in the tumor microenvironment
MDSCs	• CD11b • CD33 • CD80 • CD115 • CD124	• IL-10 • TGF-β • CCL3 • CCL4 • CCL5	• Inhibition of T cell proliferation • Recruitment of regulatory T cells • Modulation of cytokine production from macrophages during the innate immune response • Angiogenesis promotion • Tumor cell invasion and metastasis
Macrophages and Microglia	• CD45 • CD68 • CD86 • CD163 • CD204 • CD206 • iNOS	• TNFα • TGFβ • Arg1 • IFNγ	• Can be either pro (M1 macrophages) or anti-inflammatory (M2 macrophages) depending upon factors in the tumor microenvironment. • M1 Macrophages: ○ Th1 response activation ○ Phagocytosis ○ Antigen presentation • M2 Macrophages: ○ Tissue repairment ○ Hypoxia induction ○ Angiogenesis promotion
Dendritic Cells	• CD14 • CD45 • CD11c • CD31 • CD34	• IL-1 • IL-6 • IL-10 • IL-12 • IL-23	• Cancer immunosurveillance • Antigen presentation • Naïve T cell stimulation • Angiogenesis promotion

This table describes the various types of myeloid cells that have been observed in the glioma microenvironment with a description of their respective major surface markers and primary cytokines released. Additionally, we describe some of the primary effects these cells have in the tumor microenvironment.

## Myeloid cells in pediatric glioma

In a study by Lieberman et al., tumor conditioned media was collected from the cultures of patient-derived pGBM cell lines as well as other tumor types (36). These medias were used to culture DIPG cells as well as GBM cells. In co-cultures of GBM cells and healthy donor monocytes, it was observed that macrophages upregulated the anti-inflammatory markers CD163 and CD206, as well as PD-L1. In contrast, the authors noted that H3.3K27M DIPG cultures had little effect on macrophage phenotype. These findings are supported clinically as the team found that in IHC analysis of pHGGs (excluding DIPG), there was a significantly higher number of CD163^+^ macrophages in the tumor microenvironment compared to control tissue (36). However, for DIPG tissue samples, no significant increase in CD163^+^ TAMs compared to control tissue was observed (36). Furthermore, a significant increase in CD3^+^ and CD8^+^ cytotoxic T-cells was reported in pHGG tissue samples when compared to control, however, this was not observed in DIPG (36).

A study by Lin et al. observed a significant increase in the number of CD11b^+^ macrophages in the leukocyte compartment (CD45^+^) of DIPG as compared to CD3^+^ T-cells. These findings were compared to adult GBM tissue samples. Additionally, the authors note that DIPG-associated macrophages expressed fewer inflammatory cytokines (compared to those in adult GBM) in patient tissue samples, and that there is dramatically less expression of cytokines and chemokines in patient-derived DIPG cell cultures when compared to adult GBM cell cultures (35).

These studies as well as others highlight that some pHGGs, such as DIPG (an H3 mutant tumor), have limited T cell infiltration which should be considered for certain immunotherapies. However, other pHGG entities, such as pGBM, may be more immunologically active, indicating these patients may be more likely to respond to immunotherapy if the immunosuppressive milieu of the TME can be overcome, a milieu that myeloid cells largely contribute to (37, 38).

## Characterization and polarization of tumor-associated macrophages (TAMs)

In the human brain, macrophages and microglia represent a large contingent of non-neuronal cells that are myeloid cells originating from the bone marrow and yolk sac, respectively (26, 39). It has been well established that many macrophages have pro-tumorigenic effects in gliomas, and that the infiltration of these pro-tumorigenic macrophages, which are referred to as tumor-associated macrophages (TAMs), is partially driven by platelet-derived growth factor subunit B (PDGFB) signaling (26, 40). It has been observed in adult gliomas that PDGFB is highly overexpressed by macrophages and that its receptor PDGFRB is highly expressed by malignant cells (41, 42).

Previous studies have highlighted CD45, CD68, and CD163 expressing cells as being frequently expressed in the pGBM TME (26, 40). These markers are commonly expressed by TAMs and microglia (43, 44). TAMs have been observed as a predominant cell population within the TME of pGBM (26, 40). Lin and colleagues note that once TAMs become activated, they undergo a series of morphological and transcriptomic changes, such as an amoeboid morphology with shorter processes and larger cell bodies (26, 35).

Macrophages, including TAMs, are highly plastic and can undergo differentiation to alter their phenotype and function based on their local environment and concentration of signaling molecules present (45). This process, termed polarization, creates general categories of macrophages: classically activated pro-inflammatory M1-like macrophages (M1) and alternatively activated anti-inflammatory M2-like macrophages (M2) (46). Importantly, this distinction is not binary, but rather places M1-like and M2-like macrophages at opposite ends of a polarization axis in which a gradient of cell states exist with mixed characteristics of M1-like and M2-like macrophages (47).

M1-like macrophages are thought to be a part of the anti-tumor response by inducing an inflammatory microenvironment by factors such as TNF-α, IFN-y, IL-6, and CSF-2. They are characterized by markers such as HLA-DR, CD11c, CD86, iNOS, and pSTAT1, and often demonstrate IFN-y and TLR4 (LPS-induced) signaling (46, 48). M2-like macrophages are historically thought of as pro-tumorigenic due to their involvement in promoting angiogenesis, hypoxia induction, tissue repairment, anti-inflammatory TME, through expression of IL-10, TGF-β, and PGE2 and VEGF. These macrophages are classically characterized by markers such as CD163, CD204, CD206, and cMAF, and often demonstrate signaling pathways related to IL-4, IL-13, IL-10 (46, 48). M2-like macrophages can further be subdivided along a gradient depending on specific cellular markers and functionality, into M2a, M2b, M2c, and M2d macrophages (49). M2a macrophages are thought to be involved in allergic reactions, and tissue repair. M2b and M2c macrophages have been implicated in anti-inflammatory pathways and M2d macrophages have been shown to be further involved in anti-inflammation and angiogenesis (49). In pGBM and pHGG it is largely unknown whether TAMs in the TME are more M1-like vs. more M2-like, highlighting an area in need of further investigation.

## Functions of TAMs in gliomas

Previous research has highlighted how M2-like macrophages are both highly immunosuppressive, and aid in the growth of gliomas (50). TAMs can be regulated by colony stimulating factor-1 (CSF-1) for differentiation and survival. A study by Pyonteck and colleagues identified that CSF1R inhibition increased mouse survival and regressed established tumors in mice bearing patient-derived glioma xenografts (51). Additionally, the authors found that while the number of TAMs did not decrease, the number of signature markers associated with M2 phenotypes (specifically, *ADM*, *ARG1*, *F13A1*, *SERPINB2*, *MRC1*) did decrease (51). This finding highlights how CSF1R inhibition therapy impedes macrophage polarization into a pro-tumor M2 phenotype and may allow these cells to shift toward a more anti-tumor M1 phenotype (51). Another study found that PTEN deficiency in glioma enables glioma cells to secrete high levels of Galectin-9 (52). Galectin-9 is a driver of macrophage M2 polarization due to the activation of the Tim-3 receptor (52). The research team found that by blocking Galectin-9 and Tim-3 (immune-checkpoint molecules expressed on T cells and myeloid cells in adult and pediatric glioma TMEs) signaling, led to a reduction of M2 polarization and subsequent impairment in tumor progression (52–54).

A separate study by Li and colleagues found that Beta2-Microglobulin in glioma stem cells promoted an increased release of TGF-β1 from glioma cells via activation of the PI3K/AKT/MYC axis. This in turn resulted in a paracrine response of SMAD and PI3K/AKT signaling in TAMs, resulting in the polarization of TAMs toward an M2-like phenotype (55). This finding highlights an additional mechanism that drives TAMs towards an M2 phenotype, and it is crucial to explore whether Beta-2 microglobulin or its downstream axis can be therapeutically exploited in an attempt to reduce the immunosuppressive nature of the glioma TME (55). While a limited number of studies have attempted to therapeutically block or reduce polarization of M2 macrophages, the aforementioned studies highlight the diverse mechanisms that contribute to polarization of TAMs towards an M2-like phenotype. This finding highlights the need for further research that is aimed at developing therapeutics that can blockade this polarization.

Microglia are considered CNS tissue-resident macrophages. These cells make up nearly a quarter of the non-neuronal cell population within a healthy brain and provide support and protection of neuronal function (26, 39). Microglia are derived from the embryonic yolk sac, unlike macrophages which arise in the bone marrow, and maintain themselves locally in the CNS (56). While it has been demonstrated that microglia play an essential role in generating robust innate and adaptive immune responses in CNS diseases outside of brain tumors, little is known as to how microglia specifically impacts the TME in pediatric gliomas and pGBM (57). In adult GBM, it has been reported that glioma-associated microglia express anti-inflammatory markers such as CD163 and that these cells release factors that stimulate cellular migration (58, 59). Additionally, it has been shown that microglia can promote the creation of neuroblasts, allowing for the enhancement of not only neurogenesis but also oligodendrogenesis, features associated with GBM development (58, 59). Exploring the role of microglia in pHGG and pGBM is an area critically in need of investigation as microglia can have a large presence in the glioma TME, and similar to bone marrow-derived macrophages are also capable of M1/M2-like polarization (60).

## Myeloid-derived suppressor cells (MDSCs)

Myeloid-derived suppressor cells (MDSCs) were first described approximately 35 years ago as having a negative impact on the immune response during tumorigenesis (61–63). MDSCs have been characterized as having myeloid origin and a relatively immature phenotype (61–63). During normal growth and development, common myeloid progenitor cells differentiate into various types of myeloid cells (granulocytes, macrophages, dendritic cells, etc.), in the bone marrow. However, when disease states occur, there is a hindrance of normal differentiation contributing to the presence of MDSCs (61–63). MDSCs are a heterogenous population of immature myeloid cells associated with many cancers which can inhibit T-cell proliferation (64). There are ample studies that show these cells are present in adult gliomas and several studies have also shown their presence in some pHGG types, with limited knowledge in pGBM (65–70).

MDSCs express certain cell surface receptors, including CD33 (a common myeloid marker), but do not express markers of mature myeloid cells (61, 62). In mice, MDSCs are often characterized by the expression of the CD11b and Gr1 (ly6C/Ly6G) receptors depending on the phenotype of the MDSC. Ly6C and Ly6G is also often used to distinguish between myeloid type (M) and polymorphonuclear or granulocytic (PMN/G)-MDSC, respectively (64). In patients, MDSCs that are in the monocytic phenotype commonly are described by their expression as CD11b^+^CD14^+^HLA-DR^−/lo^CD15^−^, while polymorphonuclear (PMN) MDSCs are associated with CD11b^+^CD14^−^CD15^+^ or CD11b^+^CD14^−^CD66b^+^ (63). MDSCs also express multiple other surface proteins including CD80, CD115, and CD124 (61). As tumorigenesis occurs, immature myeloid progenitors develop into MDSCs with the above-mentioned characteristic cell markers and significantly increase in number. The factors that help maintain MDSCs include prostaglandins, stem-cell factors, macrophage colony-stimulating factor (M-CSF), granulocyte/macrophage colony-stimulating factor (GM-CSF), and VEGF. Through various signaling mechanisms including JAK and STAT3, MDSCs are differentiated and expand (61). A required ability of MDSCs is their ability to inhibit T-cells (61).

During tumorigenesis, it has been postulated that MDSCs have several functions: they suppress the T-cell response, modulate cytokine production from macrophages, and can advance angiogenesis, tumor cell invasion, and metastasis (61–63). As these MDSCs increase in number, they travel to the site of the tumor, likely by chemotaxis, as a result of the inflammatory response (61). The increased presence of these cells in the TME results in tumor upregulation of nitric oxide synthase activity, leading to increased amounts of nitric oxide, reactive oxygen species, and arginase 1, thus supporting an anti-inflammatory TME (61, 62, 71, 72). For instance, arginase 1 contributes to T-cell inactivation by depleting arginine, a necessary component for T-cell receptor Zeta chain expression (73).

Recently, several groups have tried to target MDSCs, whether that be by attempting to induce cellular differentiation, or block expansion, activation, and recruitment. Different research groups used docetaxel (inhibits microtubules function) and paclitaxel (chemotherapy agent) to interfere with the differentiation of myeloid cells into MDSCs (63). They demonstrated that docetaxel promoted MDSCs to differentiate toward an M1-like phenotype, whereas it was shown that paclitaxel promotes tumor MDSCs to differentiate toward a mature DC phenotype (63, 74, 75). Although this has shown some effectiveness in breast cancer, there are no studies evaluating this treatment in brain tumors (63). Other studies have tried to prevent expansion of MDSCs, specifically by targeting CSF and VEGF. Inhibition of CSF signaling have limited MDSC expansion and tumor angiogenesis (61), whereas, using anti-VEGF has been shown to significantly decrease circulating MDSC numbers (76). More so, by using amp-activated protein kinase (AMPK), to target the signaling pathways that are used by MDSCs, there was an effective decrease in the expansion and activation of MDSCs (63). Drugs which target PDGF and VEGF receptors, such as Sunitinib, have been effective in limiting the expansion and activation of MDSCs, in renal cell carcinoma (63). Metformin, (generally used for diabetes) has also shown effectiveness in decreasing the expansion and activation of MDSCs due to the drug decreasing immunosuppressive effects via the activation of AMPK (63). In mouse studies, coupling ROS inhibitors with NSAID drugs decreased the activity of MDSCs and increased antitumor activity of T-cells (61). Together, these findings suggest that therapeutic exploitation of pHGG and pGBM may be possible via the targeting of MDSCs.

## Dendritic cells

Dendritic cells (DCs) play a major role in cancer immunosurveillance as they are potent professional antigen presenting cells (APCs) which can initiate anti-tumor immune responses (77). Data shows that infiltration of DCs into primary tumor lesions is usually associated with prolonged patient survival and a reduced incidence of metastatic disease in patients with oral cancers, head and neck tumors, nasopharyngeal tumors, lung, bladder, esophageal, and gastric carcinomas (78). In their immature state, these cells can be found in a variety of non-lymphoid tissues and organs, but their activation initiates their migration to lymphoid tissues to interact with T cells and induce immune responses.

DCs have also been observed in the pGBM tumor microenvironment, however, their impact on patient prognosis as well as their exact functions within pediatric gliomas are poorly understood (79). Along this line, activation and polarization of DCs depend on the local microenvironment and can be blocked or polarized by specific factors. Therefore, the TME can result in the formation of distinct DC subsets with tolerogenic and/or immunosuppressive phenotypes (80), which could be the case in pediatric gliomas. Thus, studies of DCs in gliomas are further needed to reveal specific mechanisms these cells can employ to enable immune suppression or immune activation within adult and pediatric gliomas.

Studies in non-CNS cancers such as melanoma and ovarian carcinoma have examined the immunosuppressive role of DCs and these studies as well as others will be highlighted throughout the remainder of this section to feature potential functions of DCs in the pediatric glioma TME. One of the major immune evasion mechanisms exhibited by tumors is the expression of tumor cell-intrinsic factors. Spranger and colleagues found that in both humans and mice, melanoma tumors with active β-catenin reduce CC-chemokine ligand 4 (CCL4) expression which resulted in lower conventional DC type 1 (cDC1) infiltration and increased tumor growth (81). Conversely, other studies have observed that tumor-infiltrating natural killer (NK) cells can recruit cDC1s through production of CCL5 and XC-chemokine ligand 1 (XCL1) (82) and promote their survival with FMS-related tyrosine kinase 3 ligand (FLT3l) (83). Despite this, tumor cells can reduce NK cell viability and pro-inflammatory chemokine secretion by producing prostaglandin E2 (PGE2). This in turn limits cDC1 density and favors tumor growth (82). In addition to mitigating infiltration, the TME also curbs DC maturation, polarization, and survival through the release of vascular endothelial growth factor (VEGF), which can inhibit FLT3l activity, an essential ligand needed for cDC development and proliferation *in situ* (84). As cDC precursors are found in the TME, tumor-derived factors can locally affect pre-DC differentiation steps as well (85).

Evidence suggests DC maturation in the TME may lead to efficient priming of T-cells and benefit anti-tumor immunity in some settings (86–88). Despite this, there are molecular mechanisms that are responsible for dysfunction of DCs. Signal transducer and activator of transcription 3 (STAT3) hyperactivation can disarm DCs and subvert the protective immune surveillance of cancers (89). In addition to STAT3′s native oncogenic abilities, one aspect of its suppressive effects on DC function is related to the regulation of soluble tumor-derived factors such as VEGF and IL-10 (90, 91), both of which can be potent inhibitors of DC maturation in the TME (91–93).

Future studies should aim to determine whether these cells are immunostimulatory or dysfunctional, a potential therapeutic roll for STAT3 inhibition, and the underlining mechanisms regulating these cells in the context of pediatric glioma.

## Discussion

**Figure 1:**
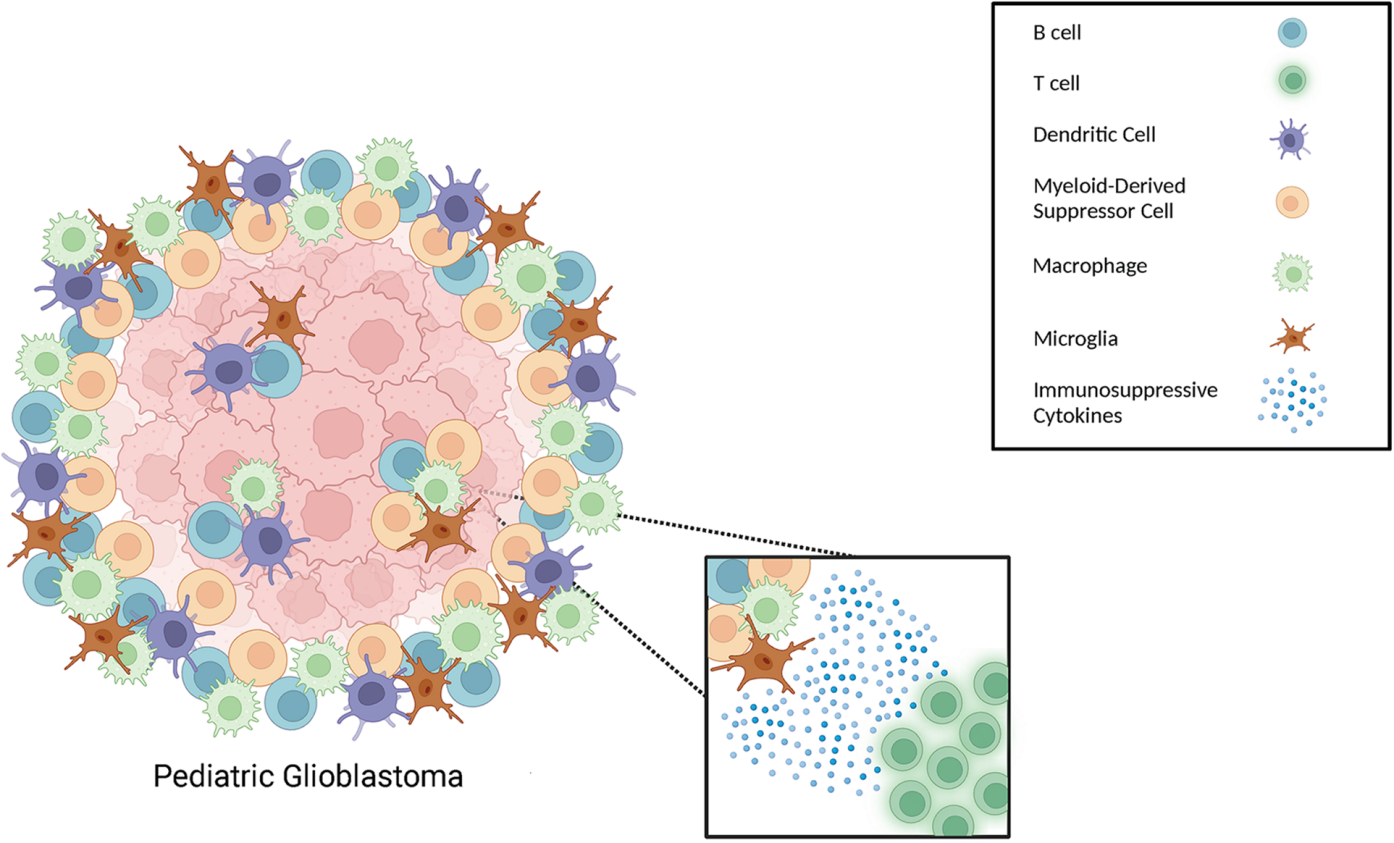
Different types of immune cells that can be present in the pediatric glioma TME. Schematic illustration of immune cells, including different types of myeloid cells, within the TME of gliomas.

In this review we highlighted the different types of myeloid cells that comprise the pHGG and pGBM microenvironment, as well as other myeloid cell types that we postulate to be relevant to the pediatric glioma TME, due to their established presence in adult gliomas. These include the presence of tumor-associated macrophages and microglia, MDSCs, and dendritic cells (see Figure 1) (79, 94–98). The role of TAMs, including macrophages and microglia, in brain tumor progression is still an emerging area of research in neuro-oncology and efforts aimed at blocking macrophage polarization to an M2 phenotype have shown promise in mouse studies. While blocking of Galectin-9/Tim-3 signaling has been shown to interfere with the polarization of macrophages toward an M2 phenotype (99–101), blocking of Galectin-9 in pediatric gliomas should be more closely evaluated. For example, studies in non-CNS cancers have shown that high expression of Galectin-9 was correlated with an improved outcome for patients diagnosed with breast cancer, melanoma, HCC, colon cancer, as well as bladder urothelial carcinoma (99, 100, 102–106). These findings highlight the need for additional studies to explore the potential effects of blocking Galectin-9, and its potential implication on TAM polarization, in patients with pHGG and pGBM (99).

MDSCs are classically recognized as being one of the major drivers of the immunosuppressive TME that is observed in CNS and non-CNS cancers. In recent years, research teams have attempted to target MDSCs as these cells are recognized as a major blockade to the success of immunotherapies. The use of docetaxel and paclitaxel to prevent differentiation of myeloid cells into MDSCs has shown promise in breast cancer, however, this approach has not been explored in brain tumors, including pediatric gliomas, and is an area in need of investigation. VEGF has been shown to be a key promoter of MDSC proliferation and expansion, and anti-VEGF has been shown to reduce the number of MDSCs within the TME and in circulation in a dose-dependent manner (76, 107). Bevacizumab (anti-VEGF monoclonal antibody) is an FDA approved therapy for the treatment of adults with recurrent GBM that have experienced disease progression following prior therapy. While this approach has demonstrated limited benefit for improving the overall and progression-free survival of (adult) GBM patients, bevacizumab may reduce the number of MDSCs in the glioma TME and this approach should be further explored (108). Furthermore, as discussed, targeting VEGF with immunotherapy could also directly inhibit glioma cells growth and maintain DC integrity and immune-promoting functions. Overall, these findings highlight why bevacizumab may be beneficial to pair with immunotherapies aimed at combatting pediatric gliomas.

Ultimately, several studies in CNS and non-CNS cancers have highlighted the increased presence of myeloid cells in the TME and how increased numbers of these cells often lead to a worsened prognosis. Additionally, several research groups have begun to therapeutically target these cell populations in non-CNS cancers in hopes of creating a TME that is less immunosuppressive, increasing the likelihood of immunotherapy success. Further research is certainly needed in these areas, as well as ways to monitor for patient response to treatment as there are limited treatment options for pGBM and pHGG. We posit that therapeutic success for these aggressive gliomas may require interfering with the immunosuppressive TME that engulfs this malignancy (109–111).

## References

[1] Arora RS Alston RD Eden TO Estlin EJ Moran A Birch JM. Age-incidence patterns of primary CNS tumors in children, adolescents, and adults in England. Neuro Oncol. (2009) 11(4):403–13. 10.1215/15228517-2008-097 19033157 PMC2743220

[2] Cosnarovici MM Cosnarovici RV Piciu D. Updates on the 2016 world health organization classification of pediatric tumors of the central nervous system—a systematic review. Med Pharm Rep. (2021) 94(3):282–8. 10.15386/mpr-1811 34430849 PMC8357361

[3] Frederico SC Zhang X Hu B Kohanbash G. Pre-clinical models for evaluating glioma targeted immunotherapies. Front Immunol. (2023) 13:1092399. 10.3389/fimmu.2022.1092399 36700223 PMC9870312

[4] Mackay A Burford A Carvalho D Izquierdo E Fazal-Salom J Taylor KR Integrated molecular meta-analysis of 1,000 pediatric high-grade and diffuse intrinsic pontine glioma. Cancer Cell. (2017) 32(4):520–37.e5. 10.1016/j.ccell.2017.08.017 28966033 PMC5637314

[5] Buccoliero AM Giunti L Moscardi S Castiglione F Provenzano A Sardi I Pediatric high grade glioma classification criteria and molecular features of a case series. Genes (Basel). (2022) 13(4):624. 10.3390/genes13040624 35456430 PMC9028123

[6] Argersinger DP Rivas SR Shah AH Jackson S Heiss JD. New developments in the pathogenesis, therapeutic targeting, and treatment of H3K27M-mutant diffuse midline glioma. Cancers (Basel). (2021) 13(21):5280. 10.3390/cancers13215280 34771443 PMC8582453

[7] Lewis PW Muller MM Koletsky MS Cordero F Lin S Banaszynski LA Inhibition of PRC2 activity by a gain-of-function H3 mutation found in pediatric glioblastoma. Science. (2013) 340(6134):857–61. 10.1126/science.1232245 23539183 PMC3951439

[8] Buczkowicz P Hoeman C Rakopoulos P Pajovic S Letourneau L Dzamba M Genomic analysis of diffuse intrinsic pontine gliomas identifies three molecular subgroups and recurrent activating ACVR1 mutations. Nat Genet. (2014) 46(5):451–6. 10.1038/ng.2936 24705254 PMC3997489

[9] Solomon DA Wood MD Tihan T Bollen AW Gupta N Phillips JJ Diffuse midline gliomas with histone H3-K27M mutation: a series of 47 cases assessing the spectrum of morphologic variation and associated genetic alterations. Brain Pathol. (2016) 26(5):569–80. 10.1111/bpa.12336 26517431 PMC6055926

[10] Bayliss J Mukherjee P Lu C Jain SU Chung C Martinez D Lowered H3K27me3 and DNA hypomethylation define poorly prognostic pediatric posterior fossa ependymomas. Sci Transl Med. (2016) 8(366):366ra161. 10.1126/scitranslmed.aah6904 PMC5123566 27881822

[11] Mohammad F Weissmann S Leblanc B Pandey DP Hojfeldt JW Comet I EZH2 Is a potential therapeutic target for H3K27M-mutant pediatric gliomas. Nat Med. (2017) 23(4):483–92. 10.1038/nm.4293 28263309

[12] Rakotomalala A Bailleul Q Savary C Arcicasa M Hamadou M Huchedé P H3.3K27M mutation controls cell growth and resistance to therapies in pediatric glioma cell lines. Cancers (Basel). (2021) 13(21):5551. 10.3390/cancers13215551 34771714 PMC8583077

[13] Tauziède-Espariat A Siegfried A Uro-Coste E Nicaise Y Castel D Sevely A Disseminated diffuse midline gliomas, H3K27-altered mimicking diffuse leptomeningeal glioneuronal tumors: a diagnostical challenge!. Acta Neuropathol Commun. (2022) 10(1):119. 10.1186/s40478-022-01419-3 35986414 PMC9392342

[14] Marlow C Cuoco JA Hoggarth AR Stump MS Apfel LS Rogers CM. Pediatric diffuse hemispheric glioma H3 G34-mutant with gains of the BRAF locus: an illustrative case. Rare Tumors. (2023) 15:20363613231168704. 10.1177/20363613231168704 37056711 PMC10088409

[15] Singla AK Madan R Gupta K Goyal S Kumar N Sahoo SK Clinical behaviour and outcome in pediatric glioblastoma: current scenario. Radiat Oncol J. (2021) 39(1):72–7. 10.3857/roj.2020.00591 33794576 PMC8024182

[16] Das KK Mehrotra A Nair AP Kumar S Srivastava AK Sahu RN Pediatric glioblastoma: clinico-radiological profile and factors affecting the outcome. Child’s Nervous System. (2012) 28(12):2055–62. 10.1007/s00381-012-1890-x 22903238

[17] Ratnam NM Sonnemann HM Frederico SC Chen H Hutchinson MND Dowdy T Reversing epigenetic gene silencing to overcome immune evasion in CNS malignancies. Front Oncol. (2021) 11:719091. 10.3389/fonc.2021.719091 34336705 PMC8320893

[18] Ratnam NM Frederico SC Gonzalez JA Gilbert MR. Clinical correlates for immune checkpoint therapy: significance for CNS malignancies. Neuro-oncol Adv. (2020) 3(1):vdaa161. 10.1093/noajnl/vdaa161 PMC7813206 33506203

[19] Frederico SC Hancock JC Brettschneider EES Ratnam NM Gilbert MR Terabe M. Making a cold tumor hot: the role of vaccines in the treatment of glioblastoma. Front Oncol. (2021) 11:672508. 10.3389/fonc.2021.672508 34041034 PMC8141615

[20] Akindona FA Frederico SC Hancock JC Gilbert MR. Exploring the origin of the cancer stem cell niche and its role in anti-angiogenic treatment for glioblastoma. Front Oncol. (2022) 12:947634. 10.3389/fonc.2022.947634 36091174 PMC9454306

[21] Frederico SC Darling C Bielanin JP Dubinsky AC Zhang X Hadjipanayis CG Neoadjuvant immune checkpoint inhibition in the management of glioblastoma: exploring a new frontier. Front Immunol. (2023) 14:1057567. 10.3389/fimmu.2023.1057567 36875096 PMC9981631

[22] Das KK Kumar R. Pediatric glioblastoma. In: De Vleeschouwer S, editor. Glioblastoma. Brisbane, AU: Codon Publications Copyright: The Authors (2017). 10.15586/codon.glioblastoma.2017.ch15 29251872

[23] Wyss J Frank NA Soleman J Scheinemann K. Novel pharmacological treatment options in pediatric glioblastoma—a systematic review. Cancers (Basel). (2022) 14(11):2814. 10.3390/cancers14112814 35681794 PMC9179254

[24] Shalita C Hanzlik E Kaplan S Thompson EM. Immunotherapy for the treatment of pediatric brain tumors: a narrative review. Transl Pediatr. (2022) 11(12):2040–56. 10.21037/tp-22-86 36643672 PMC9834947

[25] Frederico S Sneiderman C Pollack I Kohanbash G. Developing an adoptive cell transfer immunotherapy for pediatric high-grade gliomas. J Immunother Cancer. (2022) 10(Suppl 2):A236-A. 10.1136/jitc-2022-SITC2022.0222

[26] Price G Bouras A Hambardzumyan D Hadjipanayis CG. Current knowledge on the immune microenvironment and emerging immunotherapies in diffuse midline glioma. EBioMedicine. (2021) 69:103453. 10.1016/j.ebiom.2021.103453 34157482 PMC8220552

[27] Griesinger AM Birks DK Donson AM Amani V Hoffman LM Waziri A Characterization of distinct immunophenotypes across pediatric brain tumor types. J Immunol. (2013) 191(9):4880–8. 10.4049/jimmunol.1301966 24078694 PMC3827919

[28] De Leo A Ugolini A Veglia F. Myeloid cells in glioblastoma microenvironment. Cells. (2020) 10(1):18. 10.3390/cells10010018 33374253 PMC7824606

[29] Lin Y-J Wu CY-J Wu JY Lim M. The role of myeloid cells in GBM immunosuppression. Front Immunol. (2022) 13:887781. 10.3389/fimmu.2022.887781 35711434 PMC9192945

[30] Müller S Kohanbash G Liu SJ Alvarado B Carrera D Bhaduri A Single-cell profiling of human gliomas reveals macrophage ontogeny as a basis for regional differences in macrophage activation in the tumor microenvironment. Genome Biol. (2017) 18(1):234. 10.1186/s13059-017-1362-4 29262845 PMC5738907

[31] Gomez Perdiguero E Klapproth K Schulz C Busch K Azzoni E Crozet L Tissue-resident macrophages originate from yolk-sac-derived erythro-myeloid progenitors. Nature. (2015) 518(7540):547–51. 10.1038/nature13989 25470051 PMC5997177

[32] Ginhoux F Prinz M. Origin of microglia: current concepts and past controversies. Cold Spring Harb Perspect Biol. (2015) 7(8):a020537. 10.1101/cshperspect.a020537 26134003 PMC4526747

[33] De Kleer I Willems F Lambrecht B Goriely S. Ontogeny of myeloid cells. Front Immunol. (2014) 5:423. 10.3389/fimmu.2014.00423 25232355 PMC4153297

[34] Caronni N Savino B Bonecchi R. Myeloid cells in cancer-related inflammation. Immunobiology. (2015) 220(2):249–53. 10.1016/j.imbio.2014.10.001 25454487

[35] Lin GL Nagaraja S Filbin MG Suvà ML Vogel H Monje M. Non-inflammatory tumor microenvironment of diffuse intrinsic pontine glioma. Acta Neuropathol Commun. (2018) 6(1):51. 10.1186/s40478-018-0553-x 29954445 PMC6022714

[36] Lieberman NAP DeGolier K Kovar HM Davis A Hoglund V Stevens J Characterization of the immune microenvironment of diffuse intrinsic pontine glioma: implications for development of immunotherapy. Neuro Oncol. (2019) 21(1):83–94. 10.1093/neuonc/noy145 30169876 PMC6303470

[37] Njonkou R Jackson CM Woodworth GF Hersh DS. Pediatric glioblastoma: mechanisms of immune evasion and potential therapeutic opportunities. Cancer Immunol Immunother. (2022) 71(8):1813–22. 10.1007/s00262-021-03131-y 35020009 PMC10992220

[38] Wang Z Guo X Gao L Wang Y Guo Y Xing B Classification of pediatric gliomas based on immunological profiling: implications for immunotherapy strategies. Mol Ther Oncolytics. (2021) 20:34–47. 10.1016/j.omto.2020.12.012 33575469 PMC7851498

[39] Guillemin GJ Brew BJ. Microglia, macrophages, perivascular macrophages, and pericytes: a review of function and identification. J Leukocyte Biol. (2004) 75(3):388–97. 10.1189/jlb.0303114 14612429

[40] Ross JL Chen Z Herting CJ Grabovska Y Szulzewsky F Puigdelloses M Platelet-derived growth factor beta is a potent inflammatory driver in paediatric high-grade glioma. Brain. (2020) 144(1):53–69. 10.1093/brain/awaa382 PMC7954387 33300045

[41] Hara T Chanoch-Myers R Mathewson ND Myskiw C Atta L Bussema L Interactions between cancer cells and immune cells drive transitions to mesenchymal-like states in glioblastoma. Cancer Cell. (2021) 39(6):779–92.e11. 10.1016/j.ccell.2021.05.002 34087162 PMC8366750

[42] Kohanbash G McKaveney K Sakaki M Ueda R Mintz AH Amankulor N GM-CSF promotes the immunosuppressive activity of glioma-infiltrating myeloid cells through interleukin-4 receptor-α. Cancer Res. (2013) 73(21):6413–23. 10.1158/0008-5472.CAN-12-4124 24030977 PMC3829000

[43] Jurga AM Paleczna M Kuter KZ. Overview of general and discriminating markers of differential microglia phenotypes. Front Cell Neurosci. (2020) 14:198. 10.3389/fncel.2020.00198 32848611 PMC7424058

[44] Han X Liu Y-J Liu B-W Ma Z-L Xia T-J Gu X-P. TREM2 And CD163 ameliorate microglia-mediated inflammatory environment in the aging brain. J Mol Neurosci. (2022) 72(5):1075–84. 10.1007/s12031-022-01965-4 35306602

[45] Pan Y Yu Y Wang X Zhang T. Tumor-associated macrophages in tumor immunity. Front Immunol. (2020) 11:583084. 10.3389/fimmu.2020.583084 33365025 PMC7751482

[46] Yunna C Mengru H Lei W Weidong C. Macrophage M1/M2 polarization. Eur J Pharmacol. (2020) 877:173090. 10.1016/j.ejphar.2020.173090 32234529

[47] Italiani P Boraschi D. From monocytes to M1/M2 macrophages: phenotypical vs. Functional differentiation. Front Immunol. (2014) 5:514. 10.3389/fimmu.2014.00514 25368618 PMC4201108

[48] Orecchioni M Ghosheh Y Pramod AB Ley K. Macrophage polarization: different gene signatures in M1(LPS+) vs. Classically and M2(LPS-) vs. Alternatively activated macrophages. Front Immunol. (2019) 10:1084. 10.3389/fimmu.2019.01084 31178859 PMC6543837

[49] Roszer T. Understanding the mysterious M2 macrophage through activation markers and effector mechanisms. Mediators Inflamm. (2015) 2015:816460. 10.1155/2015/816460 26089604 PMC4452191

[50] Vidyarthi A Agnihotri T Khan N Singh S Tewari MK Radotra BD Predominance of M2 macrophages in gliomas leads to the suppression of local and systemic immunity. Cancer Immunol Immunother. (2019) 68(12):1995–2004. 10.1007/s00262-019-02423-8 31690954 PMC11028103

[51] Pyonteck SM Akkari L Schuhmacher AJ Bowman RL Sevenich L Quail DF CSF-1R inhibition alters macrophage polarization and blocks glioma progression. Nat Med. (2013) 19(10):1264–72. 10.1038/nm.3337 24056773 PMC3840724

[52] Ni X Wu W Sun X Ma J Yu Z He X Interrogating glioma-M2 macrophage interactions identifies gal-9/tim-3 as a viable target against PTEN-null glioblastoma. Sci Adv. (2022) 8(27):eabl5165. 10.1126/sciadv.abl5165 35857445 PMC9269888

[53] Li G Wang Z Zhang C Liu X Cai J Wang Z Molecular and clinical characterization of TIM-3 in glioma through 1,024 samples. Oncoimmunology. (2017) 6(8):e1328339. 10.1080/2162402X.2017.1328339 28919992 PMC5593703

[54] Ausejo-Mauleon I Labiano S de la Nava D Laspidea V Zalacain M Marrodán L TIM-3 blockade in diffuse intrinsic pontine glioma models promotes tumor regression and antitumor immune memory. Cancer Cell. (2023) 41(11):1911–26.e8. 10.1016/j.ccell.2023.09.001 37802053 PMC10644900

[55] Li D Zhang Q Li L Chen K Yang J Dixit D *β*2-Microglobulin maintains glioblastoma stem cells and induces M2-like polarization of tumor-associated macrophages. Cancer Res. (2022) 82(18):3321–34. 10.1158/0008-5472.CAN-22-0507 35841593

[56] Greter M Lelios I Croxford AL. Microglia versus myeloid cell Nomenclature during brain inflammation. Front Immunol. (2015) 6:249. 10.3389/fimmu.2015.00249 26074918 PMC4443742

[57] Yang I Han SJ Kaur G Crane C Parsa AT. The role of microglia in central nervous system immunity and glioma immunology. J Clin Neurosci. (2010) 17(1):6–10. 10.1016/j.jocn.2009.05.006 19926287 PMC3786731

[58] Geribaldi-Doldán N Fernández-Ponce C Quiroz RN Sánchez-Gomar I Escorcia LG Velásquez EP The role of microglia in glioblastoma. Front Oncol. (2021) 10:603495. 10.3389/fonc.2020.603495 33585220 PMC7879977

[59] Matias D Balça-Silva J da Graça GC Wanjiru CM Macharia LW Nascimento CP Microglia/astrocytes-glioblastoma crosstalk: crucial molecular mechanisms and microenvironmental factors. Front Cell Neurosci. (2018) 12:235. 10.3389/fncel.2018.00235 30123112 PMC6086063

[60] Orihuela R McPherson CA Harry GJ. Microglial M1/M2 polarization and metabolic states. Br J Pharmacol. (2016) 173(4):649–65. 10.1111/bph.13139 25800044 PMC4742299

[61] Gabrilovich DI Nagaraj S. Myeloid-derived suppressor cells as regulators of the immune system. Nat Rev Immunol. (2009) 9(3):162–74. 10.1038/nri2506 19197294 PMC2828349

[62] Murdoch C Muthana M Coffelt SB Lewis CE. The role of myeloid cells in the promotion of tumour angiogenesis. Nat Rev Cancer. (2008) 8(8):618–31. 10.1038/nrc2444 18633355

[63] Gao X Sui H Zhao S Gao X Su Y Qu P. Immunotherapy targeting myeloid-derived suppressor cells (MDSCs) in tumor microenvironment. Front Immunol. (2020) 11:585214. 10.3389/fimmu.2020.585214 33613512 PMC7889583

[64] Raphael I Kumar R McCarl LH Shoger K Wang L Sandlesh P TIGIT and PD-1 immune checkpoint pathways are associated with patient outcome and anti-tumor immunity in glioblastoma. Front Immunol. (2021) 12:637146. 10.3389/fimmu.2021.637146 34025646 PMC8137816

[65] Alban TJ Bayik D Otvos B Rabljenovic A Leng L Jia-Shiun L Glioblastoma myeloid-derived suppressor cell subsets express differential macrophage migration inhibitory factor receptor profiles that can be targeted to reduce immune suppression. Front Immunol. (2020) 11:1191. 10.3389/fimmu.2020.01191 32625208 PMC7315581

[66] Mi Y Guo N Luan J Cheng J Hu Z Jiang P The emerging role of myeloid-derived suppressor cells in the glioma immune suppressive microenvironment. Front Immunol. (2020) 11:737. 10.3389/fimmu.2020.00737 32391020 PMC7193311

[67] Raychaudhuri B Rayman P Ireland J Ko J Rini B Borden EC Myeloid-derived suppressor cell accumulation and function in patients with newly diagnosed glioblastoma. Neuro-Oncology. (2011) 13(6):591–9. 10.1093/neuonc/nor042 21636707 PMC3107102

[68] Toonen JA Solga AC Ma Y Gutmann DH. Estrogen activation of microglia underlies the sexually dimorphic differences in Nf1 optic glioma-induced retinal pathology. J Exp Med. (2017) 214(1):17–25. 10.1084/jem.20160447 27923908 PMC5206494

[69] Bayik D Zhou Y Park C Hong C Vail D Silver DJ Myeloid-derived suppressor cell subsets drive glioblastoma growth in a sex-specific manner. Cancer Discov. (2020) 10(8):1210–25. 10.1158/2159-8290.CD-19-1355 32300059 PMC7415660

[70] Mueller S Taitt JM Villanueva-Meyer JE Bonner ER Nejo T Lulla RR Mass cytometry detects H3.3K27M-specific vaccine responses in diffuse midline glioma. J Clin Invest. (2020) 130(12):6325–37. 10.1172/JCI140378 32817593 PMC7685729

[71] Kusmartsev S Gabrilovich DI. Inhibition of myeloid cell differentiation in cancer: the role of reactive oxygen species. J Leukoc Biol. (2003) 74(2):186–96. 10.1189/jlb.0103010 12885935

[72] Ochoa AC Zea AH Hernandez C Rodriguez PC. Arginase, prostaglandins, and myeloid-derived suppressor cells in renal cell carcinoma. Clin Cancer Res. (2007) 13(2 Pt 2):721s–6s. 10.1158/1078-0432.CCR-06-2197 17255300

[73] Taheri F Ochoa JB Faghiri Z Culotta K Park HJ Lan MS L-Arginine regulates the expression of the T-cell receptor zeta chain (CD3zeta) in jurkat cells. Clin Cancer Res. (2001) 7(3 Suppl):958s–65s. 11300497

[74] Kodumudi KN Woan K Gilvary DL Sahakian E Wei S Djeu JY. A novel chemoimmunomodulating property of docetaxel: suppression of myeloid-derived suppressor cells in tumor bearers. Clin Cancer Res. (2010) 16(18):4583–94. 10.1158/1078-0432.CCR-10-0733 20702612 PMC3874864

[75] Michels T Shurin GV Naiditch H Sevko A Umansky V Shurin MR. Paclitaxel promotes differentiation of myeloid-derived suppressor cells into dendritic cells in vitro in a TLR4-independent manner. J Immunotoxicol. (2012) 9(3):292–300. 10.3109/1547691X.2011.642418 22283566 PMC3386478

[76] Li K Shi H Zhang B Ou X Ma Q Chen Y Myeloid-derived suppressor cells as immunosuppressive regulators and therapeutic targets in cancer. Signal Transduct Target Ther. (2021) 6(1):362. 10.1038/s41392-021-00670-9 34620838 PMC8497485

[77] Shurin MR. Dendritic cells presenting tumor antigen. Cancer Immunol Immunother. (1996) 43(3):158–64. 10.1007/s002620050317 9001569

[78] Lotze MT. Getting to the source: dendritic cells as therapeutic reagents for the treatment of patients with cancer. Ann Surg. (1997) 226(1):1–5. 10.1097/00000658-199707000-00001 9242331 PMC1190900

[79] Bailey CP Wang R Figueroa M Zhang S Wang L Chandra J. Computational immune infiltration analysis of pediatric high-grade gliomas (pHGGs) reveals differences in immunosuppression and prognosis by tumor location. Comput Syst Oncol. (2021) 1(3):e1016. 10.1002/cso2.1016 34723252 PMC8553216

[80] Steinman RM Hawiger D Nussenzweig MC. Tolerogenic dendritic cells. Annu Rev Immunol. (2003) 21:685–711. 10.1146/annurev.immunol.21.120601.141040 12615891

[81] Spranger S Bao R Gajewski TF. Melanoma-intrinsic β-catenin signalling prevents anti-tumour immunity. Nature. (2015) 523(7559):231–5. 10.1038/nature14404 25970248

[82] Böttcher JP Bonavita E Chakravarty P Blees H Cabeza-Cabrerizo M Sammicheli S NK Cells stimulate recruitment of cDC1 into the tumor microenvironment promoting cancer immune control. Cell. (2018) 172(5):1022–37.e14. 10.1016/j.cell.2018.01.004 29429633 PMC5847168

[83] Barry KC Hsu J Broz ML Cueto FJ Binnewies M Combes AJ A natural killer-dendritic cell axis defines checkpoint therapy-responsive tumor microenvironments. Nat Med. (2018) 24(8):1178–91. 10.1038/s41591-018-0085-8 29942093 PMC6475503

[84] Salmon H Idoyaga J Rahman A Leboeuf M Remark R Jordan S Expansion and activation of CD103(+) dendritic cell progenitors at the tumor site enhances tumor responses to therapeutic PD-L1 and BRAF inhibition. Immunity. (2016) 44(4):924–38. 10.1016/j.immuni.2016.03.012 27096321 PMC4980762

[85] Wculek SK Cueto FJ Mujal AM Melero I Krummel MF Sancho D. Dendritic cells in cancer immunology and immunotherapy. Nat Rev Immunol. (2020) 20(1):7–24. 10.1038/s41577-019-0210-z 31467405

[86] Galon J Costes A Sanchez-Cabo F Kirilovsky A Mlecnik B Lagorce-Pagès C Type, density, and location of immune cells within human colorectal tumors predict clinical outcome. Science. (2006) 313(5795):1960–4. 10.1126/science.1129139 17008531

[87] Zhang L Conejo-Garcia JR Katsaros D Gimotty PA Massobrio M Regnani G Intratumoral T cells, recurrence, and survival in epithelial ovarian cancer. N Engl J Med. (2003) 348(3):203–13. 10.1056/NEJMoa020177 12529460

[88] Finak G Bertos N Pepin F Sadekova S Souleimanova M Zhao H Stromal gene expression predicts clinical outcome in breast cancer. Nat Med. (2008) 14(5):518–27. 10.1038/nm1764 18438415

[89] Lin A Schildknecht A Nguyen LT Ohashi PS. Dendritic cells integrate signals from the tumor microenvironment to modulate immunity and tumor growth. Immunol Lett. (2010) 127(2):77–84. 10.1016/j.imlet.2009.09.003 19778555

[90] Niu G Wright KL Huang M Song L Haura E Turkson J Constitutive Stat3 activity up-regulates VEGF expression and tumor angiogenesis. Oncogene. (2002) 21(13):2000–8. 10.1038/sj.onc.1205260 11960372

[91] Wang T Niu G Kortylewski M Burdelya L Shain K Zhang S Regulation of the innate and adaptive immune responses by stat-3 signaling in tumor cells. Nat Med. (2004) 10(1):48–54. 10.1038/nm976 14702634

[92] Han Z Dong Y Lu J Yang F Zheng Y Yang H. Role of hypoxia in inhibiting dendritic cells by VEGF signaling in tumor microenvironments: mechanism and application. Am J Cancer Res. (2021) 11(8):3777–93. 34522449 PMC8414384

[93] Schülke S. Induction of interleukin-10 producing dendritic cells as a tool to suppress allergen-specific T helper 2 responses. Front Immunol. (2018) 9:455. 10.3389/fimmu.2018.00455 29616018 PMC5867300

[94] Messiaen J Jacobs SA De Smet F. The tumor micro-environment in pediatric glioma: friend or foe? Front Immunol. (2023) 14:1227126. 10.3389/fimmu.2023.1227126 37901250 PMC10611473

[95] Ross JL Velazquez Vega J Plant A MacDonald TJ Becher OJ Hambardzumyan D. Tumour immune landscape of paediatric high-grade gliomas. Brain. (2021) 144(9):2594–609. 10.1093/brain/awab155 33856022 PMC8536940

[96] Rajendran S Hu Y Canella A Peterson C Gross A Cam M Single-cell RNA sequencing reveals immunosuppressive myeloid cell diversity during malignant progression in a murine model of glioma. Cell Rep. (2023) 42(3):112197. 10.1016/j.celrep.2023.112197 36871221

[97] Engler JR Robinson AE Smirnov I Hodgson JG Berger MS Gupta N Increased microglia/macrophage gene expression in a subset of adult and pediatric astrocytomas. PLoS One. (2012) 7(8):e43339. 10.1371/journal.pone.0043339 22937035 PMC3425586

[98] Jones C Karajannis MA Jones DTW Kieran MW Monje M Baker SJ Pediatric high-grade glioma: biologically and clinically in need of new thinking. Neuro-Oncology. (2016) 19(2):153–61. 10.1093/neuonc/now101 PMC5464243 27282398

[99] Acharya N Sabatos-Peyton C Anderson AC. Tim-3 finds its place in the cancer immunotherapy landscape. J Immunother Cancer. (2020) 8(1):e000911. 10.1136/jitc-2020-000911 32601081 PMC7326247

[100] Yamauchi A Kontani K Kihara M Nishi N Yokomise H Hirashima M. Galectin-9, a novel prognostic factor with antimetastatic potential in breast cancer. Breast J. (2006) 12(5 Suppl 2):S196–200. 10.1111/j.1075-122X.2006.00334.x 16959001

[101] Fujita K Iwama H Sakamoto T Okura R Kobayashi K Takano J Galectin-9 suppresses the growth of hepatocellular carcinoma via apoptosis in vitro and in vivo. Int J Oncol. (2015) 46(6):2419–30. 10.3892/ijo.2015.2941 25823465

[102] Holtan SG Mansfield AS Creedon DJ Nevala WK Haluska P Leontovich AA An organ system-based approach to prognosis in advanced melanoma. Front Biosci Elite. (2012) 4(8):2723–33. 10.2741/e586 22652681

[103] Zhang ZY Dong JH Chen YW Wang XQ Li CH Wang J Galectin-9 acts as a prognostic factor with antimetastatic potential in hepatocellular carcinoma. Asian Pac J Cancer Prev. (2012) 13(6):2503–9. 10.7314/APJCP.2012.13.6.2503 22938412

[104] Gu C Wu H Sheng C Ni Q. Expression and prognostic value of galectin-9 in hepatocellular carcinoma patients. Zhonghua yi xue za zhi. (2013) 93(26):2025–8. 24169278

[105] Sideras K Biermann K Verheij J Takkenberg BR Mancham S Hansen BE PD-L1, galectin-9 and CD8 + tumor-infiltrating lymphocytes are associated with survival in hepatocellular carcinoma. Oncoimmunology. (2017) 6(2):e1273309. 10.1080/2162402X.2016.1273309 28344887 PMC5353918

[106] Wang Y Sun J Ma C Gao W Song B Xue H Reduced expression of galectin-9 contributes to a poor outcome in colon cancer by inhibiting NK cell chemotaxis partially through the rho/ROCK1 signaling pathway. PLoS One. (2016) 11(3):e0152599. 10.1371/journal.pone.0152599 27028892 PMC4814049

[107] Draghiciu O Nijman HW Hoogeboom BN Meijerhof T Daemen T. Sunitinib depletes myeloid-derived suppressor cells and synergizes with a cancer vaccine to enhance antigen-specific immune responses and tumor eradication. Oncoimmunology. (2015) 4(3):e989764. 10.4161/2162402X.2014.989764 25949902 PMC4404834

[108] Gilbert MR Dignam JJ Armstrong TS Wefel JS Blumenthal DT Vogelbaum MA A randomized trial of bevacizumab for newly diagnosed glioblastoma. N Engl J Med. (2014) 370(8):699–708. 10.1056/NEJMoa1308573 24552317 PMC4201043

[109] Frederico SC Vera E Abdullaev Z Acquaye A Aldape K Boris L Heterogeneous clinicopathological findings and patient-reported outcomes in adults with MN1-altered CNS tumors: a case report and systematic literature review. Front Oncol. (2023) 13:1099618. 10.3389/fonc.2023.1099618 36741001 PMC9892899

[110] Penas-Prado M Yuan Y Wall K Vera E Ikiddeh-Barnes U Blackburn K CTIM-32. Immune checkpoint inhibitor nivolumab in people with recurrent select rare CNS cancers: results of interim analysis in a heavily pretreated cohort. Neuro Oncol. (2021) 23(Suppl 6):vi57–8. 10.1093/neuonc/noab196.224

[111] Frederico SC Darling C Zhang X Huq S Agnihotri S Gardner PA Circulating tumor DNA—a potential aid in the management of chordomas. Front Oncol. (2022) 12:1016385. 10.3389/fonc.2022.1016385 36338734 PMC9632974

